# Disability competency training in medical education

**DOI:** 10.1080/10872981.2023.2207773

**Published:** 2023-05-06

**Authors:** Danbi Lee, Samantha W. Pollack, Tracy Mroz, Bianca K. Frogner, Susan M. Skillman

**Affiliations:** a Department of Rehabilitation Medicine, University of Washington, Seattle, USA; b Center for Health Workforce Studies, Department of Family Medicine, University of Washington, Seattle, USA

**Keywords:** Disability competency, disability, diversity, medical education, health care education

## Abstract

Purpose. Lack of health care providers’ knowledge about the experience and needs of individuals with disabilities contribute to health care disparities experienced by people with disabilities. Using the Core Competencies on Disability for Health Care Education, this mixed methods study aimed to explore the extent the Core Competencies are addressed in medical education programs and the facilitators and barriers to expanding curricular integration. Method. Mixed-methods design with an online survey and individual qualitative interviews was used. An online survey was distributed to U.S. medical schools. Semi-structured qualitative interviews were conducted via Zoom with five key informants. Survey data were analyzed using descriptive statistics. Qualitative data were analyzed using thematic analysis. Results. Fourteen medical schools responded to the survey. Many schools reported addressing most of the Core Competencies. The extent of disability competency training varied across medical programs with the majority showing limited opportunities for in depth understanding of disability. Most schools had some, although limited, engagement with people with disabilities. Having faculty champions was the most frequent facilitator and lack of time in the curriculum was the most significant barrier to integrating more learning activities. Qualitative interviews provided more insight on the influence of the curricular structure and time and the importance of faculty champion and resources. Conclusions. Findings support the need for better integration of disability competency training woven throughout medical school curriculum to encourage in-depth understanding about disability. Formal inclusion of the Core Competencies into the Liaison Committee on Medical Education standards can help ensure that disability competency training does not rely on champions or resources.

## Introduction

People with disabilities experience persistent disparities in health status and health care. [1, 2,3] Central to the multilevel barriers to adequate health care are the lack of awareness and training among health care providers about the varied experiences and needs of individuals with disabilities, as well as negative attitudes and assumptions [4,5]. People with disabilities continue to experience challenges in accessing quality health care because of lack of disability-competent care by providers such as inaccessible space and equipment, poor communication, and not considering existing disability or functional status in making treatment decisions [6–8]. A recent study of over 25,000 health care providers, including physicians, found that more than 60% were unaware of their own implicit bias against people with disabilities [9]. Yet, literature shows that providers receive only limited training addressing negative attitudes towards disability and the wide range of health care needs of people with disabilities including specific clinical and access needs [10–13].

A review of published U.S. medical school disability curricula in 2017 found that the level of integrating disability competency remained to be heterogeneous and primarily exposure-based with only a few schools providing a longitudinal model [12]. These range from didactic methods like lectures and single courses, to the inclusion of standardized patients with disabilities during clerkship rotations; 6-week integrated clerkship experiences; and 4-year integrated disability curriculum that included all of the above modalities and the option of attending a 4^th^-year disability-focused elective clerkship. The variability in delivering disability curricula also extends to content as there has been lack of agreement on what to teach about disability in medical education [14].

Recognizing this gap, in 2019, the Alliance for Disability for Health Care Education published Core Competencies on Disability for Health Care Education (Core Competencies) to promote the integration of disability-related content and experiences into health care education and training programs [15]. Members of the alliance composed of health care educators, faculty and professionals across different health care disciplines drafted the competencies and received feedback through a two-wave iterative process from 140 disability experts and health educators. This two-year process resulted in six core competencies and 49 sub-competencies (Table 1) that provide health care education standards on social, environmental, and physical aspects of disability [14,15]. There has yet been a study that examined whether and how these Core Competencies are addressed in medical education. The study aimed to explore the extent the Core Competencies are addressed in medical education programs in the U.S. and the facilitators and barriers to expanding curricular integration.

**Table 1.** Description of the Core Competencies^15^.

**Competency 1 (contextual and conceptual frameworks)** reframes the concept of disability from a health outcome to a patient characteristic through a conceptual framework that considers the lifespan, sociocultural factors, and individual factors into a patient’s experience with disability. There are six sub-competencies that break this down by including definitions of disability – within civil rights (1.4) and social determinants of health (1.5)—as well as considering the diversity of disability (1.1) and the historical context and cultural identity of disability (1.6). **Competency 2 (professionalism and patient-centered care)** directly addresses the biases against people with disabilities and how to overcome them through professionalism, communication, and respect. The nine sub-competencies achieve this through confronting one’s own implicit biases (2.1), demonstrations of communication (2.3) and patient-centered care (2.4), as well as understanding how a patient’s diverse cultural identity (2.7), social determinants of health (2.8), and specific disability needs (2.9) can affect the provision of care. **Competency 3 (legal requirements and responsibilities)** reviews the legal acts and requirements (i.e., Americans with Disabilities Act) for providing care to patients with disabilities that meets their needs, such as physical requirements (3.2), communication requirements (3.3, 3.4), and being aware of other possible barriers to accessing the healthcare system as a patient with a disability (3.1, 3.7, 3.8). **Competency 4 (team-based** **care)** promotes team collaboration and emphasizes the importance of interprofessional teams to address the sometimes complex needs of patients with disabilities and providing them with the highest quality care. The five sub-competencies recognize the different models of team-based care and members of the team (4.1, 4.2), strategies to build a healthcare team and address challenges (4.3), demonstrating the necessary skills for working with a healthcare team (4.4), and understanding issues patients may face in systems-based care and appropriate solutions, such as community-based resources (4.5). **Competency 5 (clinical assessment)** covers the need for consideration of a patient’s functional status when clinically assessing them and considering their activities and goals when working directly with the patient to develop a patient-specific care plan. There are thirteen sub-competencies that outline how to best provide clinical care to people with disabilities, including listening to the patient and consulting with a caregiver only when necessary (5.1, 5.2) and incorporating specific patient information for clinical decision making such as functional status (5.3, 5.5), gender, language, race, ethnicity, sexual orientation (5.4), other health issues associated with the disability (5.7), variability of the disability over time (5.8), possible co-occurring mental health conditions and misdiagnoses (5.10), social environment and possible exposure to abuse (5.11, 5.12), and physical environment (5.13). **Competency 6 (clinical care over the lifespan)** addresses the importance of incorporating how transitions across the life span should be taken into account for clinical care and care coordination. This includes being sensitive to a patient’s changing health care needs throughout different life stages (6.1) and considering age-appropriate health screenings and treatments for reproductive health (6.3), as well as being able to identify and recommend community resources (6.5) and interprofessional health care providers (6.7) for health education and promoting healthy behaviors.

## Methods

The study used a sequential mixed-methods design, where an online survey was followed by qualitative interviews. Both quantitative and qualitative data were collected to provide a more in-depth and complete understanding of the problem [16]. This study was determined to be exempt by the University of Washington Institutional Review Board (IRB# MOD00007591).

### Questionnaire

A 23-item questionnaire was developed by a multidisciplinary project team of experts in the research areas of disability studies (DL), health services (TM, BF, SS, SP), and health workforce (DL, TM, BF, SS, SP). Multiple-choice questions were used to identify which Core Competencies are currently addressed in the curriculum; what facilitators and barriers schools experience in to incorporating disability content into the curriculum; and how people with disabilities are involved. Open-ended questions were used to gather details of learning activities mapped to the Core Competencies (i.e., name, content/topic, format, required/optional, and timing of the activity). Preliminary survey questions were pilot-tested and reviewed by an advisory panel of six experts including faculty from medical schools with expertise in disability education and experts with disabilities from disability organizations advocating for equitable health care of people with disabilities. The survey was then revised based on their feedback.

The survey was distributed to all allopathic and osteopathic medical schools in the U.S. (*n* = 196), including programs with preliminary or provisional accreditation status as of the 2019 academic year. Email invitations and six reminders were sent to curriculum deans, deans of undergraduate education, and program directors between February and June 2020. Research Electronic Data Capture (REDCap) was used for data collection of survey responses [17].

### Qualitative interviews

Qualitative interviewees were purposefully selected from survey respondents to represent medical schools from different regions. Thirty to sixty-minute individual interviews were conducted by the first and second authors via Zoom using a semi-structured interview guide. Informed by the survey findings, the interview guide that was designed to better understand the learning activities and barriers and supports described in the survey (Table 2). The interview guide was developed by the same multidisciplinary project team. Prior to the interview, participants provided informed consent to their research participation. Consented participants were asked to describe learning activities with disability content and their impact on students, facilitators to initiating and maintaining learning activities, and barriers to integrating more. Interviews were recorded with permission.

**Table 2.** Questions from the qualitative interview guide.

Please describe any disability related learning activities your program provides. What changes, if any, have you noticed/observed with your students who went through the learning activities? What impact do you think the learning activities had on students? When and how did you start those learning activities? What resources did/would you need to sustain these learning activities in your program? What would you like to add/change in the future? What prevents you/your program from having the above mentioned (or more) disability-related activities in the curriculum? Please tell me what would help you/your program incorporate more disability-related activities in the curriculum.

### Data analysis

Following the data triangulation protocol suggested by O’Cathain et al. [18], data were first analyzed separately then integrated at the interpretation stage. First, survey data were analyzed using descriptive statistics to identify which and how many Core Competencies were addressed in medical schools, how people with disabilities are involved, and what supports and barriers exist. Details of the learning activities described in the open-ended questions were coded (e.g., types, focus, length, and frequency of the learning activities) and summarized qualitatively and quantitatively. Then, notes and transcripts from the individual interviews were analyzed using thematic analysis [19]. Themes were discussed with the research team. Credibility, transferability, and confirmability were ensured through coding-recoding, data triangulation, thick description of data, and reflexivity (i.e., constant reflection and discussion regarding positionality as persons without disabilities). Finally, to add depth to the interpretation, results from quantitative and qualitative data were compared and examined for convergence, complementarity, and discrepancy [18].

## Results

### Participants

A total of 14 programs completed the survey. Most respondents were allopathic public medical schools with larger cohorts (Table 3). Five medical school representatives participated in the qualitative interview. This included one private and four public medical schools representing four U.S. census regions.

**Table 3.** Institutional characteristics of participants.

Survey Respondents (*n* = 14)					Count (%)
Degree type		MD			11 (78.6)
		DO			3 (21.4)
Census region		South			7 (50.0)
		Midwest			3 (21.4)
		Northeast			2 (14.3)
		West			2 (14.3)
Cohort size		100+ students			11 (78.6)
		75–99 students			2 (14.3)
		50–74 students			1 (7.1)
Institution type		Public			10 (71.4)
		Private			4 (28.6)
Faculty/student disability status		Faculty with disclosed or visible disability (yes)			8 (61.5)
		Current students with disclosed or visible disability (yes)			11 (84.6)
Qualitative Interviewees (*n* = 5)					
	Degree	Region	Cohort size	Type	Survey Respondent
CS1	MD	Northeast	100+	Public	Yes
CS2	MD	Midwest	100+	Public	Yes
CS3	MD	South	100+	Public	Yes
CS4	MD	West	100+	Public	No
CS5	MD	West	75–99	Private	No

### Findings

Integrated, the results from the survey and qualitative interviews were categorized into two topic areas: 1) status of disability competency training addressing the Core Competencies and 2) barriers and facilitators to integrating disability competency training. Qualitative data particularly provided more in-depth understanding on 1) the influence of curricular structure and time on integrating Core Competencies and 2) the crucial role of resources and champions.

### Status of disability competency training addressing the Core Competencies

Eleven out of 14 schools reported that their curriculum addresses five to six Core Competencies in their curriculum (Table 4). Most schools (*n* = 13) said that they address contextual and conceptual frameworks on disability and teams and systems-based practice. Competencies around legal obligations and responsibilities were least addressed (*n* = 6).

**Table 4.** Core Competencies addressed in learning activities.

Characteristics	Count (%)	Examples
**Core Competencies addressed through learning activities (*n* = 14**)		
Competency 1: Contextual and conceptual frameworks on disability	13 (92.9)	Lectures on medical and social model of disability, disability and culture, challenges living in an ableist society, and disability activism.
Competency 4: Teams and systems-based practice	13 (92.9)	Interprofessional education event with medical students and nurses; lab with interdisciplinary activities.
Competency 2: Professionalism and patient-centered care	12 (85.7)	Patient panel; bias training; etiquette and disability language
Competency 5: Clinical assessment	11 (78.6)	History and Physical examination simulations with patients with disabilities or standardized patients
Competency 6: Clinical care over the lifespan and during transitions	9 (64.3)	Field project with people with disabilities in rural communities; clinical rotations in geriatrics; clinical experience with children and adults with intellectual and developmental disabilities.
Competency 3: Legal obligations and responsibilities	7 (50)	Discussion on disability within a historical and legal context; health care disparities; and community resources
**Number of Disability Core Competencies addressed by schools (*n* = 14)**		
Six Disability Core Competencies	6 (42.9%)	
Five Disability Core Competencies	5 (35.7%)	
Four Disability Core Competencies	1 (0.07%)	
One Disability Core Competency	2 (14.3%)	

The extent of disability competency training varied. About half of the medical schools reported one or two learning activities within their curriculum; the other half described three or more learning activities. Most learning activities described were offered in single 45-minute to 2-hour sessions such as one-time patient panels or patient simulations. Some were longer and more integrated across different courses and extended time periods, including integrated cases over 2 years, weekly simulations over a year, and 4-week clinical rotations. The majority of learning activities were required although most of the extended experiences such as advanced placement or clinical rotations were optional. Except for the clinical rotations in year 4 and a few patient encounters in year 3, all learning activities reported were completed during the first two years of medical education.

The learning activities included lectures, case studies, panel discussions, and small group discussions. Many discussed topics such as different disability models, ableism, and implicit bias to raise awareness of disability; addressed disability etiquette and how to interact with people with disabilities in clinical assessments or in interdisciplinary care; and provided opportunities to learn about the lived experiences of people with disabilities through patient panels. Some schools reported activities focused on learning about disability as a medical condition within the context of rehabilitation (e.g., visiting rehabilitation sites, clinical rotation in PM&R) or understanding disability-related diagnoses (e.g., cerebral palsy, dementia) in a medical context. One-time encounters and simulations with people with disabilities or standardized patients were more common, and less schools offered immersive experiential learning opportunities such as a field project with disability communities or extended clinical experiences with people with disabilities. Specific examples of learning activities linked to the Core Competencies are listed in Table 4.

Most frequently, the medical school curricula addressed physical disability (*n* = 13) while sensory disabilities were least discussed (*n* = 9). The survey result also shows that people with disabilities were often engaged in learning activities as panelists (*n* = 9) or patients (*n* = 7) with less of a role in teaching (*n* = 4) or planning curricular activities (*n* = 4). Three schools reported no involvement of individuals with disabilities. (see Table 5)

**Table 5.** Types of disabilities addressed in learning activities and engagement of patients with disabilities in learning activities.

Characteristics		Count (%)
**Types of disabilities discussed in learning activities (*n* = 13)**		
Physical disability		13 (100)
Communication (speech, language, and voice) disability		11 (84.6)
Intellectual/developmental disability		11 (84.6)
Psychiatric disability/mental health		11 (84.6)
Deaf/hard of hearing		9 (69.2)
Blind/visual impairment		9 (69.2)
**Engagement of people with disabilities in disability competency training (*n* = 12)**		
Sharing experience/knowledge in panels or lectures (as ‘peer experts’)		9 (75.0)
Interacting with students as patients (e.g., labs, role-plays, interviews)		7 (58.3)
Involved as planning/advisory members (as ‘peer educators’)		4 (33.3)
Instructor identifies as someone with a disability		4 (33.3)
Not directly involved		3 (25.0)

Similar to the survey results, in the qualitative interviews with key informants, many learning activities discussed involved short independent sessions with panels, simulations, or discussions that address particular topics. Interviewees noted that panels and patient encounters are often liked by students and have a positive impact.

… students have voiced that they feel a lot more confident after these sessions … going into the room with a patient with [disabilities], knowing how to act, and kind of owning that sometimes, yeah, you’re going to feel a little awkward, that’s ok … be mindful of etiquette and how you go about an H&P [history and physical examination], you know, ask the patient. (CS1)

However, because they were typically offered only once throughout the curriculum, many interviewees described those as not enough. Some interviewees stressed the importance of talking about disability in multiple places throughout the four years: ‘I think a better way to get more disability into the curriculum would be to put more examples of people with disabilities…peppered throughout…’ A few mentioned how they weave disability content into the diversity and health disparities discussion. One school included two disability-related learning events as part of their diversity and health care disparities thread in their 3^rd^ and 4^th^ year family medicine and internal medicine clerkships. Another interviewee shared,

We have … the autism panel and the simulated patient encounter … in a part of the curriculum where they’re talking about patients within populations … I was able to make the case that disability is totally appropriate in this context [of understanding social determinants of health] … (CS2)

Participants who only had learning activities in their first two years agreed that an iterative experience tying back the information in later clinical years is missing: ‘[T]here’s nothing in the third and fourth year where people necessarily come together to think back on what they learned in the first two years … So it’s completely hit or miss what they get’ (CS5). They also recognized that program structure could be a barrier: ‘[after the first 18 months] I don’t think there is a capitalization on the patient experience to build their knowledge in their phases of training because [students] are in hundreds of locations’ (CS4). This participant yet expressed the potential of using required online lectures to integrate disability content during clerkships.

### Barriers and facilitators to integrating disability competency training

As seen in Table 6, the most frequently identified facilitator to incorporating disability competency training into curriculum was having a faculty champion (*n* = 11) followed by support of academic leadership (*n* = 8) and partnership with community-based disabilities organizations (*n* = 7). Having students, faculty, or staff with disabilities in the program also seems to positive affect the integration of disability competency training. An overwhelming barrier was lack oftime in the curriculum to add new content (*n* = 10), followed by inadequate resources (*n* = 5). Lack of factors identified as facilitators (e.g., faculty champion, relationship with disability organizations) was also reported as barriers by some respondents.

**Table 6.** Factors influencing incorporation of disability competency training in medical school curriculum (*n* = 14).

Factors	Count (%)
**Facilitators**	
Faculty champion	11 (84.6)
Support of academic leadership	8 (61.5)
Partnership with community-based disabilities organizations	7 (53.8)
Student advocacy/champion	5 (38.5)
Faculty with a disability	3 (23.1)
Past or current student(s) with a disability	2 (15.4)
Medical school staff (non-faculty) with a disability	1 (7.7)
**Barriers**	
Lack of time in current curriculum to add new content	10 (90.9)
Inadequate resources (e.g., funding, time to restructure curriculum)	5 (45.5)
Lack of faculty to teach disability-related content	2 (18.2)
Lack of learning materials for curriculum development (e.g., books, readings, documentaries, and video sources)	2 (18.2)
Lack of relationships with community-based disability organizations (e.g., from which to recruit speakers and get curriculum input)	1 (9.1)
Limited support from leadership	0
Lack of disability organizations in your vicinity	0

Consistent with the survey result, all key informants agreed that competition for limited curriculum time is the biggest challenge to integrating more disability competency content. One interviewee said, ‘finding a foothold in the curriculum is huge. It’s really hard. You fight for your two hours.’ (CS5). In the interviews, it was also clear that having a faculty or student champion has been a force in integrating disability competency training into the curriculum. The champions were usually faculty members who were invested in this topic and who developed and carried out course materials. In one program, a student champion who has a sibling with a disability initiated an elective course where students have chances to interact with patients with disabilities, their caregivers, and other health care workers that work with people with disabilities. One interviewee described that having a course director who is a physician using a wheelchair has been a ‘wonderful asset’ because he would talk about his own disability experience as a physician (CS1).

The qualitative interviews also revealed the fragility of too much reliance on these champions. The upcoming retirement of the physician who is a wheelchair user was a concern of the interviewee because students would lose the opportunity to interact with and learn from him (CS1). This sentiment was also presented by others. One person said, ‘When [the faculty champion] left, I didn’t have a connection with anyone in rehabilitation. I lost those contacts.’ (CS4). While having a champion was seen as a facilitator, one interviewee pointed out how that is a weakness of disability competency training: I think that’s a huge weakness in this effort, that the disability training approach has always relied on champions. I have so much respect for what they [champions] are able to do … But, it’s not sustainable, and it’s not scalable … [A]s soon as the champion retires, there’s like a sell-by date on the content and the curriculum. It just cannot withstand the forces of the demands on the curriculum. (CS2)

Interviewees also pointed to the critical role of institutional supports and resources in integrating disability content in the curriculum. They provided more information on what specific resources they had access to and how they utilized those. These resources included protected time to develop content, designated staff to coordinate panel or patient encounter sessions, funds to pay panelists or standardized patients and families, and connection to disability organizations to recruit patient volunteers.

Resource needs for implementing patient encounters and panels have been frequently noted. One program had many of their staff involved in supporting the panel/simulation session to ensure accessibility of participants (e.g., appropriate environment for patients with autism, guiding patients with visual impairment) (CS3). For some, the lack of resources prevented activities they believed to be best practice: ‘I brought people [with disabilities] together for the facilitators for [problem-based learning]…[H]aven’t done it since because of limited resources.’ (CS5).

Finding the right panelists for the hoped impact was sometimes a challenge. ‘I don’t know what they are going to say’ said one participant (CS4) as some panelists had other messages they wanted to communicate (e.g., gun control). When finding time for planning a panel session became a challenge due to the pandemic, this program replaced it with watching and reflecting on the ‘Crip Camp’ documentary, as a way to expose students to the lived experience of people with disabilities and the disability rights movement. The interviewee shared, ‘maybe a very well-done film will be more effective in bringing across the messages.’

Some interviewees mentioned the importance of having partnership with a disability organization or content experts from different disciplines (e.g., faculty from speech language pathology and physical therapy), as they did not have expertise in this topic. So they [clerkship directors] felt like it [disability content] was important and … probably felt a lot more confident knowing that [the disability organization] was in their corner, because they’re the experts. (CS1)

As another way to secure time and resources to develop new content, one interviewee actively sought external funding. This person reported that external grant helps ‘get a foot in the door.’ Once content is developed, those activities are typically popular with students and tend to continue after the grant ends, said this interviewee, but they would not happen in the first place without external funding: ‘Offering grants, even if it’s not a huge amount of money, is night and day in terms of getting cooperation from the medical school, and in terms of actually developing content and implementing.’ (CS2)

One interviewee suggested that embedding the Core Competencies into the Liaison Committee on Medical Education (LCME) accreditation standards may lead to proactive integration of disability content even without a champion or resources.

## Discussion

The study explored the extent medical schools integrate the Core Competencies in their curriculum and the barriers and facilitators to the integration. In survey responses, many schools reported addressing most of the Core Competencies. The extent of disability competency training varied across medical programs with the majority showing limited opportunities for in-depth understanding of disability. Most schools had some, although limited, engagement with people with disabilities. Having faculty champions was the most frequent facilitator and lack of time in the curriculum was the most significant barrier to integrating more learning activities. Qualitative interviews provided more insight on the influence of the curricular structure and time and the importance of faculty champion and resources.

Consistent with previous literature [11,12], the format and length of disability competency learning activities in medical schools varied. Although most participants in this study reported that their curriculum addresses multiple Core Competencies, most competencies were addressed in one or two learning activities that is likely not providing an in-depth understanding of the related topics. While one-time panels or patient encounters can have an impact on student confidence in interacting with people with disabilities and their understanding of disability experiences, previous research found that this impact is short term [11,20] and does not translate into improved quality of clinical care for people with disabilities long term [16]. Especially, with health care provider’s implicit bias being a contributor to equal and quality healthcare for people with disabilities, frequent opportunities to reflect on and be exposed to disability issues are critical [9]. The survey also showed that most activities were completed in the first two years. It is positive that disability content was introduced early; however, competencies related to clinical care would require students to apply their knowledge in clinical context, which often occur in later years.

As suggested by the study participants and literature, to promote long-lasting transformative experiences, medical programs should consider longitudinal, iterative, and integrated learning activities woven throughout the curriculum [8,13,20]. Along with lectures, panels, and discussions, immersive experiential learning activities would be ideal [8,21]. However, considering limited resources and time constraints, weaving content into existing curriculum and modifying existing cases in the curriculum may be more realistic and effective changes to make to integrate disability content throughout and to facilitate students’ competency in disability related clinical care [20]. Having disability representation throughout cases and normalizing talking about disability in context of diversity and cultural humility could help future physicians actively reflect on and work towards eliminating their explicit and implicit biases when working with people with disabilities [22]. Disability is often omitted from diversity and cultural competency discussions [22]. Better systemization, such as explicitly including disability as part the cultural competency in LCME standards, needs to be made for medical programs to view disability competency as an essential part of medical education and make the investment for improving curricular [22].

This study and previous publications have highlighted the need for identifying a champion to integrate disability competency training into medical education [8,13]. The dependency on champions has been identified as a contributor to variability in disability training across medical schools [14]. The lack of faculty with disability studies expertise or lived experiences across medical schools is problematic because even with a strengthened LCME standard, the ability to teach disability competency will vary. The variability may be reduced with more efforts in creating and sharing lesson plans or resources that can be easily implemented by non-experts. For example, Borowsky et al. published a participatory 2-hour lesson plan that discusses ableism, the social model of disability, disability history and culture, and health disparities [23]. With all materials needed, these plans and guides may be easy to implement for those with less knowledge or experience. Yet, most importantly, the incompetency of majority of educators in the topic of disability and lack of champions call for the critical need for diversifying the workforce by making medical education and practice more accessible and inclusive for individuals with disabilities [22].

In this study, people with disabilities were primarily involved as panelists or standardized patients rather than advisory members or instructors. Patient encounters were more often completed with individuals with disabilities and their families than with standardized actors, an approach criticized by disability communities [21]. However, we found that engaging people with disabilities or the disability community requires time and monetary and human resources and thus is often perceived as burden. In addition, connections with local disability communities are not always established without a champion. Like one school in this study shared, using documentaries or memoirs about disability rights and culture could be good alternatives for programs lacking the resources to coordinate direct involvement of people with disabilities [21]. There are also published materials on how to find and engage with local disability organizations to plan and teach disability content if a non-expert is interested in initiating a relationship [24].

Only a few schools had a person with a disability serving as an advisory member or instructor. Overall, improving disability representation among faculty, students, and advisory members will ensure that curricular decisions reflect their voices [10]. This is consistent with recent studies that confirmed the underrepresentation of disability in Medicine as having only 3.1% of physicians [25] and 4.5% of medical students identify as disabled [26]. Many barriers and needs to disability competency training could be mended with more students, faculty, and physicians with disabilities in the field. With more individuals with disabilities, there will be more champions. In addition, the interaction with someone with a disability as a peer, colleague, teacher, and mentor, and not as a patient in a clinical setting, will allow faculty and students to see the person and not their disability and debunk inaccurate assumptions and discomfort [27]. These changes can only be achieved with intentional efforts to remove barriers and promote access and inclusion in policies and practices in medical education [22,28].

This study has a few limitations. Despite efforts to recruit, the low response rate to the survey was likely influenced by the onset of the COVID-19 pandemic, which affected medical schools and directors during the study period. Schools who are more invested and interested in this topic may have been more likely to respond to the survey. In addition, schools that are not directly addressing these competencies may not have been as willing to reveal this deficit. Therefore, the study results may not represent how medical schools in general address disability competency in the curricula.

Despite these limitations, the findings allowed for an understanding of efforts made and the potential for integrating disability competency training within time-restricted medical education. It is also important to note that not all medical schools have the institutional support and champions that this study described. As recommended by one of the interviewees, further consideration of explicitly integrating the Core Competencies into LCME accreditation standards may be needed so medical schools are incentivized to integrate this important topic in their education regardless of available champions, resources, and supports. Ensuring that all physicians are trained to work with people with disabilities would be a critical step towards reducing disparities in health care for and the health of people with disabilities.
